# Fibrinaloid Microclots and Atrial Fibrillation

**DOI:** 10.3390/biomedicines12040891

**Published:** 2024-04-17

**Authors:** Douglas B. Kell, Gregory Y. H. Lip, Etheresia Pretorius

**Affiliations:** 1 Department of Biochemistry, Cell and Systems Biology, Institute of Systems, Molecular and Integrative Biology, Faculty of Health and Life Sciences, University of Liverpool, Crown St, Liverpool L69 7ZB, UK; 2 The Novo Nordisk Foundation Center for Biosustainability, Technical University of Denmark, Søltofts Plads, Building 220, 2800 Kongens Lyngby, Denmark; 3 Department of Physiological Sciences, Faculty of Science, Stellenbosch University, Private Bag X1 Matieland, Stellenbosch 7602, South Africa; 4 Liverpool Centre for Cardiovascular Science at University of Liverpool, Liverpool John Moores University and Liverpool Heart and Chest Hospital, Liverpool L7 8TX, UK; gregory.lip@liv.ac.uk; 5 Danish Center for Health Services Research, Department of Clinical Medicine, Aalborg University, 9220 Aalborg, Denmark

**Keywords:** atrial fibrillation, fibrinaloid microclots, inflammation, infection, Long COVID, particulate matter, pollution, arrhythmias

## Abstract

Atrial fibrillation (AF) is a comorbidity of a variety of other chronic, inflammatory diseases for which fibrinaloid microclots are a known accompaniment (and in some cases, a cause, with a mechanistic basis). Clots are, of course, a well-known *consequence* of atrial fibrillation. We here ask the question whether the fibrinaloid microclots seen in plasma or serum may in fact also be a cause of (or contributor to) the development of AF. We consider known ‘risk factors’ for AF, and in particular, exogenous stimuli such as infection and air pollution by particulates, both of which are known to cause AF. The external accompaniments of both bacterial (lipopolysaccharide and lipoteichoic acids) and viral (SARS-CoV-2 spike protein) infections are known to stimulate fibrinaloid microclots when added in vitro, and fibrinaloid microclots, as with other amyloid proteins, can be cytotoxic, both by inducing hypoxia/reperfusion and by other means. Strokes and thromboembolisms are also common consequences of AF. Consequently, taking a systems approach, we review the considerable evidence in detail, which leads us to suggest that it is likely that microclots may well have an aetiological role in the development of AF. This has significant mechanistic and therapeutic implications.

## 1. Introduction

Atrial fibrillation (AF) is at once the commonest heart arrhythmia [1,2,3] and a chief cause of death therefrom [4,5]. Its global incidence amounts to millions of individuals [1], possibly 1% of populations, and even 20 years ago, its cost represented some 1% of the NHS budget, with all these numbers on a rising trend [6,7,8,9], albeit with large regional differences of incidence and/or reporting (e.g., South Asia is said to be 12× lower than North America [10]). 

AF symptoms (virtually by definition) involve [11] “A supraventricular tachyarrhythmia with uncoordinated atrial electrical activation and consequently ineffective atrial contraction. Electrocardiographic characteristics of AF include:

- Irregular R-R intervals (when atrioventricular conduction is not impaired),
- Absence of distinct repeating P waves, and
- Irregular atrial activations.” [11]

Within this, there are, of course, AF subclasses with different levels of severity, especially as regards the various sequelae. AF is also associated with risk factors that rarely occur in isolation, and patients with AF are commonly associated with multimorbidity, polypharmacy, and frailty, with major implications for treatment and outcomes [12,13,14,15].

The chief fear is that AF leads to an increased likelihood of a clot forming or residing within the atrial chamber, from which it can then escape, and it is well recognised (e.g., ref. [16]) that those with AF are associated with mortality and morbidity from strokes [17,18,19,20,21,22,23,24], coronary heart disease [25,26,27] (including myocardial infarction [28]), or both [29], and vice versa, due to the build-up of blood-flow-occluding macro-clots in the relevant tissues. It should be noted, however, that because AF is often asymptomatic, and until recently was rarely screened for, many/most of these studies are actually post hoc [30,31,32,33], i.e., they study the incidence of AF in people who have had cardiovascular events rather than the other way round (i.e., for our present purposes, the incidence of heart attacks and strokes in those with known, pre-existing AF).

Our purpose here is to recognise that a certain kind of ‘fibrinaloid’ microclot that we discovered [34], despite being in many ways a ‘conventional’ blood clot formed by the polymerisation of fibrinogen to fibrin (albeit containing other proteins [35,36]), is, by virtue of its amyloid nature [37,38], rather resistant to fibrinolysis and can persist in the circulation. The presence of these microclots, commonly in the range 2–100 μm, is known to occur in a variety of conditions that may lead to or accompany AF (see below), and some of the molecules that catalyse their production such as bacterial cell wall substances [34,39,40,41,42] or the spike protein of SARS-CoV-2 [43,44] are also known. The widespread existence of these fibrinaloid microclots also led us to wonder whether they might thus contribute to the actual genesis of AF. Recognising this as a problem of systems biology [45,46,47,48], the present overview sets out what is actually considerable evidence for this view. We summarise our review in the form of a ’mind map’ [49,50]. Our approach did not use PRISMA as this was not a meta-review nor a study of an intervention.

## 2. “Risk Factors”, Covariates, Confounders, Biases, and Coherence

**Figure 1:**
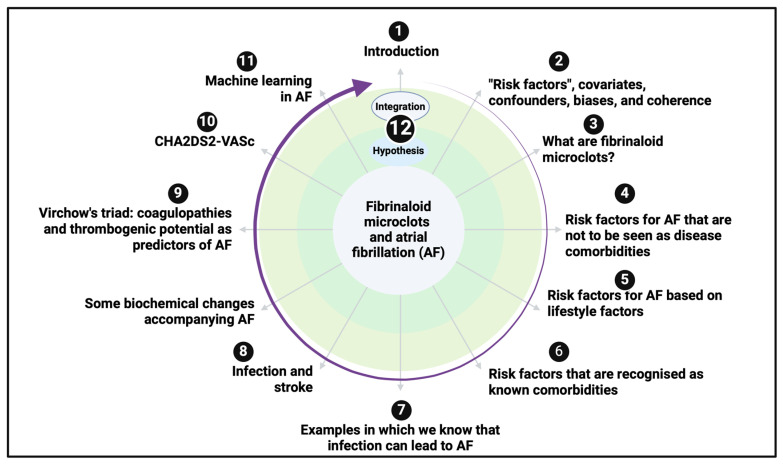
A ‘mind map’ [49] setting out this review. This is a means of summarising the review in an easy-to-visualise format. It should be read clockwise from “twelve o’clock”. Created with Biorender.com.

**Figure 2:**
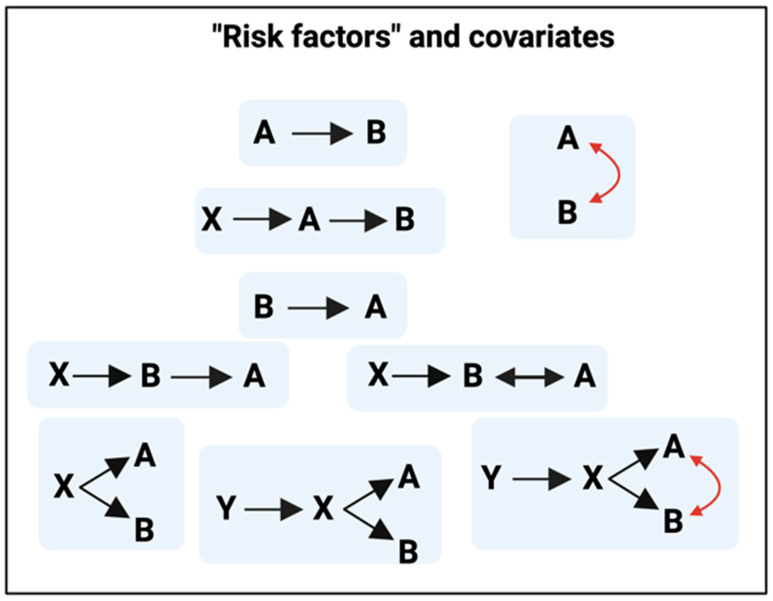
Risk factors, observables, causes, and covariates. Created with BioRender.com. The coloured arrow indicates circumstances in which A can affect B and B can affect A.

Much of modern analytics in medicine is concerned with identifying ‘risk factors’, most of which are really in fact covariates (Figure 2). To illustrate this point, we take a different cardiovascular disease (pre-eclampsia) [51,52], where, because this is a disorder of pregnancy, we do at least know the time and nature of the origin (of the pregnancy). Thus, we know that the likelihood of developing pre-eclampsia (equivalent to B in Figure 1) increases with certain pre-conditions (first pregnancy with the father, existing diabetes, maternal age, BMI, blood pressure at first visit, infection, etc.) (e.g., refs. [51,53,54,55,56,57,58,59,60]), but *a priori*, there is no way of knowing whether any of the above conditions are truly on a causal pathway or simply covariates (and caused by other factors such as those labelled X and Y in Figure 2). The infeasibility of establishing causality solely from measurements of variables is widely encapsulated in the mantra ‘correlation does not equal causation’, although any co-variation has the potential to contain useful information. Unravelling such relationships by causal inferencing either requires good longitudinal data and/or (better) affects them as independent variables. 

Thus, the fact that the long-term use of antibiotics following a toxoplasma infection lowers the risk of pre-eclampsia by a massive 11-fold [61], along with a mass of other evidence, strongly implies an infectious origin for pre-eclampsia [51,52,62], but because these are not always even recognised, they do not appear in most lists of risk factors. 

Overall, we find useful the principle of *coherence*, which indicates that if a variety of nominally orthogonal lines of evidence point towards the same mechanism, then, that mechanism is more likely to be true [63,64,65]. Similarly, we consider that an understanding of comorbidities, where the knowledge available from sets of related diseases may be brought to bear, must enhance the understanding of the disease of particular intellectual interest [66]. As Petsko [66] notes, this strategy is well established, e.g., in functional genomics (where it is often called ‘guilt by association’ (e.g., [67,68,69,70]), where it uses the idea that the co-expression of genes of unknown function with genes of known function implies a contributory role for the ‘unknown’ genes in the known function. Especially when the associations are between genetic polymorphisms and diseases, the activity—then known as genome-wide association studies or GWAS—is seen as entirely respectable [71,72,73]. Nowadays, because the statistical risks of false positives are quite considerable when the number of variables (such as SNPs) is large [74,75,76,77], the basis for any co-variation or correlation has to be very well established, and include separate validation sets. 

Consequently, although our ultimate aims are mechanistic, we shall start by looking at known ‘risk factors’ and comorbidities of AF, where the term ‘guilt by association’ has itself been used [78]. We simply note here that the terms ‘bias’ and ‘confounder’ should really just be applied in studies in which relevant and knowable covariates (e.g., gender or pre-existing pill consumption) are inadvertently distributed differently between two populations of interest (such as ‘diseased’ and ‘control’) [75]. Other kinds of ‘biases’, such as a focus on particular sets of gene products leading to models that use them, are in fact a significant feature of the now-commonplace large language models [79] based on transformers [80].

Figure 2 recognises that uncovering mechanisms in complex networks is nontrivial, a problem often encapsulated in the mantra ‘correlation does not mean causation’. More explicitly, it is common that observables A and B are seen to covary. There can be many reasons for this, e.g., that A causes B, that B causes A, that something else, X, causes them both, whether sequentially or in parallel, and/or there may be a positive feedback cycle in which A causes B and B causes A. In the present survey, A may be taken as atrial fibrillation, B as fibrinaloid microclots, X as an external effector, e.g., a viral protein such as SARS-CoV-2 spike protein or a bacterial cell wall component such as lipopolysaccharide, while Y may be inflammation/inflammatory markers. Often, we can only measure variables but it is the parameters (‘causes’) that we seek. Since we cannot always influence the parameters directly, one job of systems biology or systems medicine is to seek to reconstruct the causal relations through measurements of the variables. Longitudinal methods can here be especially powerful as a later event cannot directly influence an earlier one. We here argue that much of the evidence to date has failed to recognised the true order of events by focusing more on covariates than on longitudinal studies. 

We begin by rehearsing what we know about fibrinaloid microclots.

## 3. What Are Fibrinaloid Microclots?

**Figure 3:**
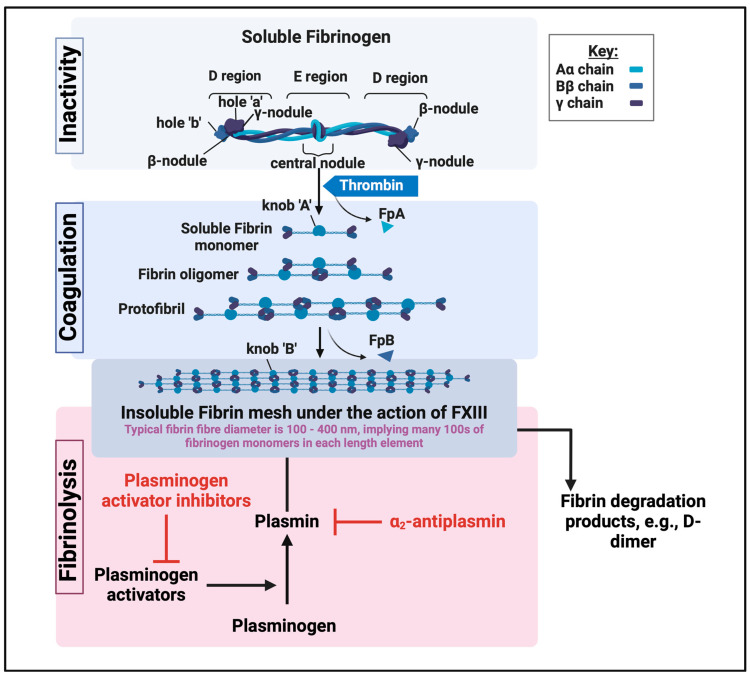
A high-level illustration of fibrinogen polymerisation into fibrin as a part of blood clotting. Created with BioRender.com. Much of the first part is redrawn from a CC-BY article at [89].

Clotting and clot removal happen all the time, so that the body is ‘primed’ for any desirable clotting to be initiated rapidly in response, say, to a wound. At a high level (Figure 3), soluble fibrinogen, typically as a 5 × 45 nm complex of three different polypeptides (α_2_β_2_γ_2_) (MW ~ 340 kDa) [81] https://www.rcsb.org/structure/3ghg plus internal fibrinopeptides A and B, and one of the most abundant proteins in plasma (present at typically 2–4 g·L^−1^ [82,83]), is acted upon by the serine protease thrombin. This action removes two fibrinopeptides, exposing ‘knobs’ and ‘holes’, and leading to a remarkable self-assembly in which fibrin monomers polymerise to make staggered oligomers, which themselves lengthen into protofibrils that aggregate laterally to make fibres, finally branching to create a three-dimensional network which represents the clots. Typical fibre diameters are a few hundred nm (say, 100–400 nm [84,85,86,87]), with a fractal morphology [88], meaning that a ‘unit’ of fibrin fibre contains many hundreds of fibrinogen monomers contributing to its diameter at any point. Clots are then degraded by plasmin, which itself has a variety of activators and inhibitors (Figure 3). For our purposes, we note that the clots may not be fully formed, that they form anomalous conformations (see below), and that their rate of degradation is, in many cases, unusually low. This means that there may be, in certain diseases, a standing crop of fibrinaloid microclots.

Fibrinolysis can be decreased by a variety of endogenous inhibitors, as well as when fibrin adopts an anomalous amyloid-type form (see below). 

**Figure 4:**
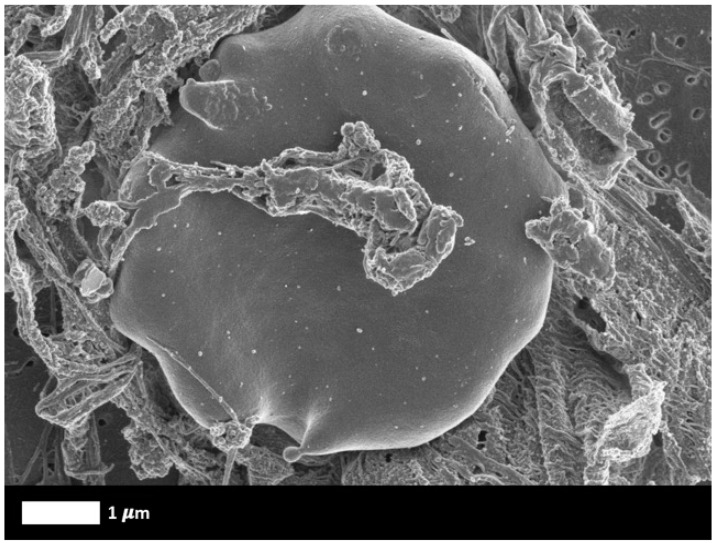
Scanning electron microscopy figure of dense matted deposits entrapping a red blood cell. Healthy whole blood was exposed to ferric chloride (raw data from [103]).

Early studies in the electron microscope by one of us (EP) showed that while images of the fibrin fibres of ‘normal’ clots looked much like nicely cooked spaghetti, those in a variety of chronic, inflammatory and other conditions looked much as if such spaghetti had been parboiled and stuck together in an unholy mess [90,91,92,93,94,95], a finding referred to at the time as ‘dense matted deposits’ (see Figure 4). These anomalous fibres could be induced by the presence of free iron [96,97,98,99,100,101,102]. 

**Figure 5:**
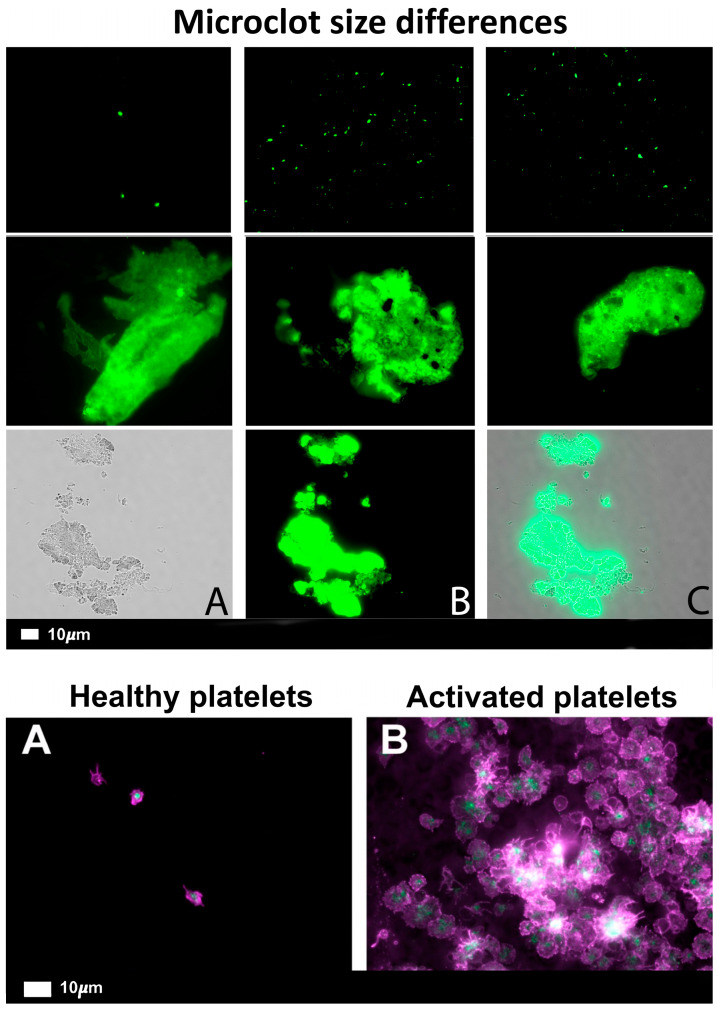
Microclots and platelets in healthy individuals and in individuals with long COVID (raw data from the CC-BY-4 publication [118]). For microclots, Figure 5 shows, in the upper three panels, the microclots observable in healthy controls at the start of the SARS-CoV-2 pandemic, while the middle three panels show microclots from individuals suffering from COVID-19. The lowest panel shows pictures from the bright field (**A**), a thioflavin T-stained image of the same field (**B**), and the merged image (**C**). For platelets, after centrifuging freshly collected samples, the haematocrit fraction of each sample was retained and incubated for 30 min at room temperature with the two fluorescent markers, CD62P (PE-conjugated) (platelet surface P-selectin) and PAC-1 (FITC-conjugated) (340507, BD Biosciences, San Jose, CA, USA). Viewing used a 63× oil objective. (**A**) Healthy controls, with minimally activated platelets, seen as small round platelets with a few pseudopodia, seen as healthy/control platelets; (**B**) diseased with egg-shaped platelets, indicative of spreading and the beginning of clumping.

Many proteins can fold into a stabler, beta-sheet-rich ‘amyloid’ form, with no change in sequence. Some of these are related to a variety of more-or-less well known diseases (‘classical amyloidoses’, e.g., refs. [104,105,106]), in which unfolded forms of proteins such as Aβ and α-synuclein are detected, but even proteins such as insulin [107] and lysozyme [108] can adopt amyloid forms. The apotheosis of this kind of behaviour is represented by prion proteins (e.g., refs. [37,38,109,110,111]), that can adopt a variety of stable, amyloid-type states (without changes in primary sequence) that can even catalyse their own (con)formation. We later showed that the ‘dense matted deposits’ were in fact amyloid in character [34,36,37,40,41,42,89,112,113,114,115,116,117], as they could be stained with the well-established amyloid stain thioflavin T, as well as the commercial ‘Amytracker’ stains [39,41,113]. This was confirmed by correlating images from the electron and fluorescence microscopes [39]. They can thus be measured in any clinical laboratory that possesses suitable fluorescence microscopes or flow cytometers. Such diseases (discussed in more detail below) were also accompanied by significant platelet activation. Figure 5 illustrates these phenomena. The thread of ideas summarised in this review goes as follows: external effectors can cause fibrin to polymerise into an amyloid-type form (Figure 4 and Figure 5 and many references cited) that is more resistant than usual to fibrinolysis, and that the fibrinaloid microclots so formed (that can aggregate into macroclots) are the cause rather than the effect of atrial fibrillation. In particular, we see this causal chain as consistent with the known ability of infections and particulate matter to lead to AF by means of what was previously an unknown mechanism.

**Figure 6:**
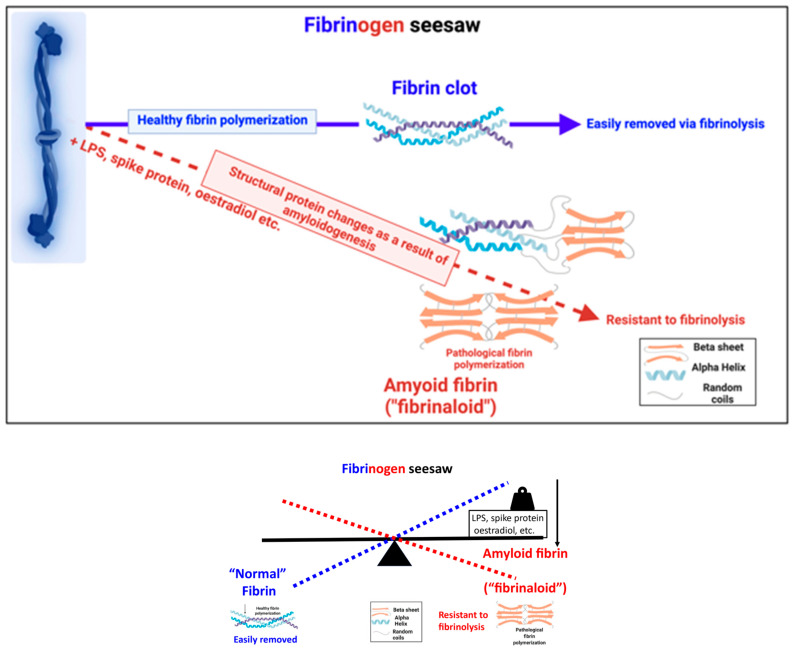
Cartoon illustrating the ability of fibrinogen to polymerise either to its α-helix-rich normal form or its crossed-β-sheet amyloid ‘fibrinaloid’ form, depending on the presence of various small-molecule triggers. Glyphs taken from the CC-BY Open Access publication [118]. Created with BioRender.com.

A particular feature of such clots is that—like prion proteins—they are far more resistant than normal to proteolysis (in this case, fibrinolysis) [37,38], and their sizes can vary widely over the main approximate range 2–100 μm diameter. The addition of known amyloids to clotting systems can induce similar effects [119]. Also, like prion proteins, these amyloid forms are thermodynamically more stable than the non-amyloid form(s) normally adopted, and the abnormal form can catalyse the transformation of the normal form into itself; consequently, this can be triggered by a minuscule amount of suitable substances, such as bacterial cell wall materials [34,41] or viral surface proteins [43]. Noting that far more small molecules bind to proteins than was widely assumed (e.g., refs. [120,121,122]), it is reasonable that any number of small molecules beyond iron ions might also effect the nature of fibrinogen polymerisation, and certainly some (such as 17-β-oestradiol [123,124]) are known to do so. Other features known to affect the rate of fibrinolysis [125] include fibre diameters [126] and the presence of anti-plasmin(ogen) proteins [35,36,127]. We illustrate the two types of fibrin as a cartoon in Figure 6.

The typical size range of these fibrinaloid microclots can allow them to travel widely through the vasculature, essentially blocking up capillaries of a suitable diameter, inhibiting blood flow and hence O_2_ transfer to tissues, and thus accounting, in principle, for the very wide range of symptoms in syndromes such as long COVID, including fatigue [89], post-exertional symptom exacerbation [128], and autoantibody induction [38]. Very recently, amyloid deposits have also been found in muscle tissues (not just plasma) of individuals with long COVID [129]. Armed with this brief summary of fibrinaloid microclots, we now turn to risk factors for atrial fibrillation.

## 4. Risk Factors for AF That Are Not to Be Seen as Disease Comorbidities

Many studies have examined how the prevalence of AF varies with properties such as age, gender, BMI, etc., that are not *per se* normally seen as comorbidities, and we shall look at them first, mostly to see if they give any hints for the incidence of microclots, before we move to the other factors (see, e.g., ref. [130], while the guidelines of Hindricks and colleagues provide a comprehensive list [11]). These few are summarised in Table 1. The focus here is on prevalence more than outcomes, since the application of therapies is not necessarily uniform [131].

Interestingly, with the possible exception of age, none of these is especially associated with AF (independently of disease) and neither is the prevalence of microclots (for instance, age and male gender do associate with acute COVID, but long COVID is far more prevalent in women (and often not the older ones)). That is already a useful test, because if there were very strong associations with ‘pure’ AF but not with microclots, it would be harder to argue for a major role of microclots in AF or vice versa. 

## 5. Risk Factors for AF Based on Lifestyle Factors

While BMI might have been placed in this category, (i) it is an effect of multiple factors as much as a cause, (ii) a high BMI can cover a multitude of physiques, such that (iii) a high BMI in a professional rugby player would not necessarily be seen as significant a risk factor as it might be in an office worker. However, other lifestyle choices are more obviously under the control of individuals, e.g., alcohol consumption, and we next look at these (Table 2). Although exposure to particulate matter may not be seen as a lifestyle choice, urban *vs.* rural living is one; particulate matter exposure (especially from cars) is far worse in the former type of living, and the effects are substantial. They also show the importance of particulate irritants, a category into which fibrinaloid microclots might be considered to fall.

Table 2 implies that within reasonable bounds, these lifestyle factors have a measurable but not massive influence on the appearance of AF in the population, and sets the scene for the discussion of the role of fibrinaloid microclots. We note that they are modifiable. 

## 6. Risk Factors That Are Recognised as Known Disease Comorbidities

Most chronic, inflammatory diseases share many properties [101], including inflammation [89] (by definition!), oxidative stress [128], and iron dysregulation [186,187,188,189] (and also, as we shall see later and in Table 3, fibrinaloid microclots). This of itself might lead one to suppose that they have a broadly similar, ultimate type of cause (i.e., something labelled “Y” in Figure 1), and the evidence for this “something” points rather squarely at an infectious origin (see Table 3). 

Apart from multiple sclerosis and sleep apnoea, that we have not yet studied, it is striking that each of the supposedly non-communicable diseases listed in Table 3 can be seen to have an infectious origin, and we know that components of both bacteria (e.g., LPS and lipoteichoic acid) and viruses (e.g., SARS-CoV-2 spike protein) can induce fibrinaloid microclots (see also below). However, our next task is to list some of these comorbidities on the grounds that they surely contain clues as to the origins of the syndrome of our present, prime focus, viz., atrial fibrillation. 

Thus, Table 4 lists some of the known comorbidities of various diseases and AF. Because of the relative lack, until recently, of long-term screening, it is not normally easy to determine longitudinal trends (potentially then causal chains), but for present purposes, an association is sufficient, before we move to cases in which we absolutely know that a specific infection can lead to AF. Table 4 makes clear that each of the diseases there also makes an appearance in Table 3, implying that they are related to each other, whether as effects of an earlier cause (as we consider likelier, Figure 1) or on their own causal chain in either direction, or both. 

## 7. Examples in Which We Know That Infection Can Lead to Atrial Fibrillation 

The conclusion from the above is that there is strong associative evidence for comorbidities of supposedly non-infectious diseases, in which fibrinaloid microclots have been demonstrated, and AF. Unfortunately, however, in most cases, a causative mechanism (or a set of mechanisms or chain of causal reasoning) is not to hand. 

However, infections represent a class of disease in which the temporal origin (of the infection) is usually known from early symptoms, so it is reasonable to ask the question whether infection is known to lead to AF in populations previously known not to manifest it. Not least since the appearance of long COVID (although there are many similar post-infection syndromes such as ME/CFS, Gulf War syndrome, post-Ebola syndrome, etc)), we have come to recognise that a variety of infections can cause major and sometimes debilitating symptoms for extended periods after the infection has nominally cleared. In the same way, pre-eclampsia is associated with a significantly increased risk of cardiovascular disease [254,255], often for many years after the pregnancy in question. 

The following facts relating infection to subsequent AF are thus highly pertinent:

- A 7.6% of cases of community acquired pneumonia led to new-onset AF [256].
- AF is a common occurrence following infection with SARS-CoV-2 (COVID-19) [257,258,259,260,261,262,263,264,265,266,267].
- The Odds Ratio (OR) for AF 365 days after COVID-19 compared to a well-established control group was 1.83 in a large study [268].
- A previous use of DOACs is protective against AF following SARS-CoV-2 infection [269]. It should be noted that AF increased the bleeding risk of those on anticoagulants [270].
- There was an increased mortality from acute COVID-19 in patients with AF [271,272,273], especially older ones.
- The same applies to long COVID [274,275] (which is not surprising, given its incidence following acute COVID).
- Cardiac arrhythmias also seem to be caused by COVID-19 vaccination [276] (which, of course, includes spike protein or RNA coding for it), and spike protein is known to cause microclots [43,44].
- New-onset AF is a common occurrence in sepsis (which, of course, convolves, e.g., infection and inflammation), increasing as sepsis leads to severe sepsis and then septic shock, leading to poorer outcomes [277,278,279,280,281,282,283,284,285,286,287,288].
- Sepsis (we ignore subtypes [289,290]) likely involves microclots [291], which can be induced experimentally in the presence of cell-surface components of infectious agents such as bacterial lipopolysaccharide [40,41,42,292] or lipoteichoic acid [41], or the spike protein of SARS-CoV-2 [43,44,293].
- Coagulation, in the worst-case disseminated intravascular coagulation, is a frequent accompaniment of sepsis [294,295,296,297,298,299,300,301].
- Anticoagulants are significantly protective against the complications of sepsis when timed properly [302,303] and especially in the presence of disseminated intravascular coagulation [304,305,306].

This raises the suspicion that coagulopathies may often precede AF, and before we look at this, we shall rehearse a few of the potentially relevant biomarkers.

## 8. Infection and Stroke

We note that stroke and infection are often associated [307,308,309,310,311], and when co-occurring, they often lead to an unfavourable outcome [312,313] (implying some co-causality). Infections associated with stroke are usually called or referred to as a ‘post-stroke infection’ (that is following a stroke), but it is equally plausible (and in some cases demonstrable [314]) that the earlier stages of infection precede the stroke event that is referred to [313]. Some of the evidence for this includes the fact that similar changes in the gut microbiome could be predictive of both stroke itself and post-stroke infection [315]. However, apart from lowering urinary tract infections, prophylactic antibiotics were mostly not preventive [316,317,318,319], cf. [320,321,322]; one interpretation of this is simply that the amount of bacterial cell wall product in the plasma necessary to induce microclots is absolute minuscule [34,41], and a tiny fraction of the total bacterial load within a person. It should, however, be noted that certain antibiotics also increase the release of such amyloidogenic bacterial cell wall materials [323,324,325,326], thereby negating (or worse) the benefits of antibiosis *per se*. 

One might also add here that the same co-association and likely causation, for which antibiotics are also not protective [327], is also true of Parkinson’s disease [328,329,330,331].

## 9. Some Biochemical Changes Accompanying AF

Given the above, it is also of interest to survey biochemical markers that might also correlate with AF. Table 5 summarises some markers that have been found to be raised in AF and that might be related to the genesis or presence of coagulopathies in general, and potentially fibrinaloid microclots.

## 10. Virchow’s Triad: Coagulopathies and Thrombogenic Potential as Predictors of AF

Although, as mentioned, AF is widely recognised as predictive of thrombus formation, our proposal is that the converse is also true (if not even more so). Is there further evidence for this? While our preferred metric is the presence of fibrinaloid microclots (measures such as D-dimer reflecting those of clot breakdown rather than clot presence [89]), these have not been carried out, so we need to look to more traditional measures of clotting potential, where similar suspicions have been raised [288,367,368,369,370], and some limited longitudinal evidence brought forward [345].

“Virchow’s triad” reflects or consists of ’abnormal blood constituents’, ’vessel wall abnormalities’ (endothelialitis), and ’abnormal blood flow’ [371], a set of coagulopathies leading to venous thrombosis, that also occur in AF [21,23,369,372,373,374,375,376,377,378,379,380], and indeed are common in both acute [381,382,383,384,385,386,387,388] and long [389,390,391] COVID (which, of course, are also characterised by fibrinaloid microclots). It should again be noted that the special feature of these fibrinaloid microclots not only makes them easy to see but makes them significantly more resistant to the normal means of fibrinolysis. This again points strongly to the potential for microclots as being causative in AF and not merely a consequence. Similarly, it is easy to suppose that microclots are potentially able to aggregate into the better known macroclots; it seems highly desirable to test these as to whether or not they are amyloid in character. 

## 11. Clinical Risk Scores, e.g., CHA_2_DS_2_-VASc

From the point of view of risk factor analysis, the main present assessment for predicting the risk of stroke in individuals known to have AF is known as (and leads to) clinical risk scores, such as the CHA_2_ DS_2_-VASc score [158], a backronym related to **C**ongestive Heart Failure, **H**ypertension, **A**ge (>75 y) (2 points), **D**iabetes Mellitus, prior **S**troke or TIA or thromboembolism (2 points), **V**ascular disease, **A**ge (again, 65–74 y), and **S**ex **c**ategory (female). The elements not marked as two points score one point, and age is either one or two points, giving a maximum score of 9. These are now in wide common use in guidelines globally [392,393]. 

**Figure 7:**
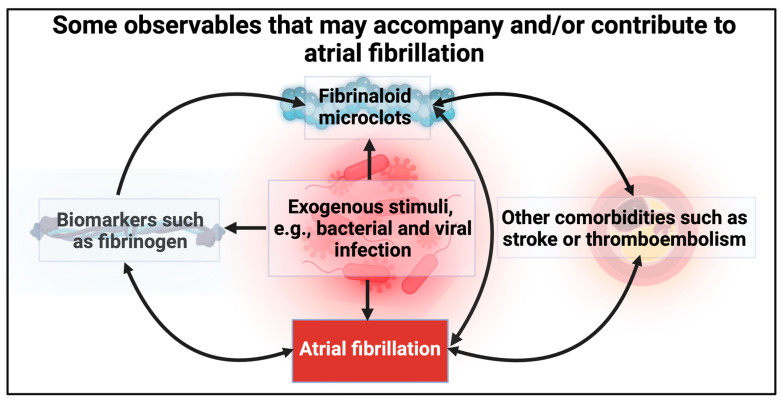
Some of the features on which we have focused in this review, indicating those (indicated by thick arrows) for which there is well-established evidence, plus others that we consider likely but for which further evidence needs to be sought. Created with BioRender.com.

However, notwithstanding that strokes are clearly caused by microclots, as phrased by Qureshi et al. [23], these scores “rely mostly on clinical comorbidities, rather than thrombogenic mechanisms such as blood stasis, hypercoagulability and endothelial dysfunction—known as Virchow’s triad.” In view of the above arguments, it does seem very timely to revisit these, and indeed to develop new methods of assessment based on markers of thrombotic problems including biochemical markers such as those in Table 5, more physiological methods of endothelial dysfunction such as flow-mediated dilatation [378], and, in particular, fibrinaloid microclots. Given the tendency of unfolded/amyloid proteins to nucleate and increase in size (e.g., refs. [394,395,396,397,398,399,400,401,402]), it is highly plausible that microclots may serve as precursors to macroclots. Figure 7 provides a summary and overview of the kinds of evidence that we have brought together here.

Specifically, if microclots themselves lead to AF (as do other particulates, see above), one mechanism is their known membrane activities that would more or less directly cause channelopathies, which are themselves known to be causative in AF, especially when inherited [403,404,405,406,407]. In a similar vein, therapeutic options for physicians to consider might include ramped-up versions of anticoagulation/anti-platelet drugs such as the ‘triple treatment’ [408], while there is also a clear role for fibrinolytic enzymes [89,128]; some of these, such as nattokinase and serrapeptase, are readily available.

## 12. Machine Learning in AF

As we move towards a post-genomic, data-driven biology [409], it is increasingly recognised that hypothesis-free methods can play a valuable role in deconstructing complex phenomena. Thus, within the AF field, machine learning has been applied to the predictive risk of many inputs [410,411], including more narrowly on ECGs [412,413] and in COVID-19 [414]. In one study, 15% of AF patients assigned to the AF cohort by the algorithm had a secondary care diagnosis with no record of AF in primary care [415]. Implementing such algorithms would be highly cost-effective [416]. Specifically, as a data-driven strategy, this section invites those measuring AF, which is nowadays carried out as routine, to also look for fibrinaloid microclots (since presently this is not the case).

## 13. Final Discussion and Conclusions, and a Forward Look

Although we have focused here on AF, we recognise that many other cardiac and (more generally) cardiovascular disorders are also accompanied by thromboses of various kinds. These include type 2 diabetes [417,418], heart failure [419], myocarditis [420], peripheral arterial disease [421], various disorders of pregnancy [422], and others that are not always (but should be) seen to be associated with vascular problems, such as Alzheimer’s and Parkinson’s [423]. Cardiac amyloidoses [424,425] are of especial interest here. The multifaceted clinical complexity of patients with AF has led to the current overall holistic or integrated care management approach to AF care [426,427], as is recommended in guidelines [393].

Learning from this collective of syndromes allows one to see common factors, at least one of which involves fibrinaloid microclots. Up to now, these have not been studied as an independent risk factor for AF. This clearly needs to change. A very striking recent example [183] shows the role of particulate nanoplastics in atheromas and subsequent cardiac events, and it would be surprising if this were not also true for AF. While present initial and successful therapies for AF focus on anticoagulation [428], the recognition of fibrinaloid microclots as a cause and not just a consequence of AF may have significant and further therapeutic implications.

Since the acceptance of this article, three significant papers on fibrinaloid microclots have been published. One [429] describes the importance of microclots in intensive care unit morbidity and disseminated intravascular coagulation, while two others describe an automated microscope method for the detection of microclots [430,431] and their degradation by nattokinase [431]. 

## Figures and Tables

**Table 1:** Variation in the prevalence of AF with certain properties of individuals.

Risk Factor	Comments	Selected References
Age	Normally a significant increase in AF with age.	[11,130,132,133]
	In some cases the opposite can be true for athletes.	[134,135]
	May involve age-dependent Na^+^ channel expression.	[136]
BMI	Obesity is sometimes a clear risk factor [4] (as for many metabolic diseases), but there are also (and more commonly) a variety of so-called ‘obesity paradoxes’, where the hazard ratios for acquiring or manifesting AF, and, in particular, suffering disease sequelae therefrom, are actually significantly greater for those with a lower BMI.	[137,138,139,140,141,142,143,144,145,146,147,148,149]
	Obesity may induce sleep apnoea, which is a known risk factor for AF.	[78,150,151,152]
Ethnicity	More prevalent among Caucasians; not entirely clear how much is genetics, culture/lifestyle, or GxE, and as with genetics, no studies really seek to deconvolve these factors.	[130]
Familial associations/Genetics	Mostly less significant than lifestyle factors and co-morbidities, apart from some particular and relatively uncommon ion channelopathies.	[153];
	Monozygotic/dizygotic ratio does predict a role for genetics, so not purely cultural associations.	[154,155]
	Highly polygenic, with genes involved in developmental, contractile, and electrophysiological functions. Necessarily convolved with GxE association that cannot be interpreted from GWAS studies alone.	[149,156,157]
Gender	More prevalent in males, though outcomes can be worse for females (so being female contributes to the CHA_2_DS_2_-VASc score [158,159]). Less important than age for asymptomatic AF.	[130,160,161]

**Table 2:** Variation in the prevalence of AF with certain ‘lifestyle’ risk factors.

Risk Factor	Comments	Selected References
Alcohol consumption	Some increase in AF risk as a function of alcohol intake; greater in men; studies mainly not controlled for BMI. Not a huge effect for moderate levels of consumption.	[145,162,163]
Exercise	As with BMI, the relationship is nonlinear, with moderate exercise and general cardio-respiratory fitness being beneficial, but excess exercise (which could cause oxidative stress [164,165], inflammation [166,167], hypoxaemia [168], etc.) having negative effects.	[145,169,170]
Particulate matter exposure	New-onset AF can follow exposure to particulate matter.	[171,172,173,174]
	This can be an acute occurrence	[175]
	Increases with known genetic risk factors.	
	Meta-analyses.	[176,177,178]
	Particulate matter is also amyloidogenic.	[179,180,181,182]
	Particulate matter can cause other cardiac problems.	[183]
Psychosocial Stress	As estimated by surrogates reflecting anger and hostility, it can be a minor risk factor in men but not women, even after controlling for hypertension.	[145,184]
Smoking	Although important to other cardiovascular diseases, for AF seemingly a marginal risk, and probably dwarfed by other risks of smoking such as lung cancer.	[185]

**Table 3:** Some supposedly non-communicable diseases for which there is, in fact, substantial evidence of an infectious element and/or evidence of fibrinaloid microclots.

Disease or Syndrome	Comments	References Regarding an Infectious Origin	References Illustrating Anomalous Clotting/Microclots
Alzheimer’s	Many references, not least from Ruth Itzhaki focusing on HSV, imply this strongly. Other organisms have also been implicated.	[190,191,192,193,194]	[39,42,100]
Diabetes, type 2	Originally asked by Gundersen in 1927. Even greater evidence for type 1 [195], not covered here.	[196,197,198,199,200]	[39,95,113,115,116,201,202]
	Many reviews (also those with T2D are more susceptible to infections; this direction is not discussed here).	[203,204,205]	
	Increased diabetes prevalence following COVID-19 infection.	[206]	
Multiple sclerosis	Now recognised as being caused by Epstein–Barr virus.	[207,208,209]	Not yet studied
Myalgic encephalitis/chronic fatigue syndrome (ME/CFS)	Clear infectious origin, likely viral, and most likely a herpes virus.	[210]	[210,211]
Parkinson’s	Induction of disease progression by bacterial LPS and by viruses.	[212,213]	[39]
	Reviews	[214,215]	
Rheumatoid arthritis	Absolutely clear evidence for *Proteus* spp. as the infectious agent.	[216,217,218,219]	[128,220,221,222]
Sleep apnoea	Obstructive sleep apnoea is a strong risk factor or comorbidity of AF, also associated with obesity [5,78,152,223] and both acute [224] and long COVID [225].	[226,227,228]	Not yet studied

**Table 4:** Some diseases or comorbidities known to be associated with AF.

Disease	Comments	Selected References
Alzheimer’s	AF is of course related to age, as is AD. Stroke is also related to vascular dementia. Strong comorbidities between cardiovascular disease and AD.	[229] [230]
	Some indications that AF is associated with an exacerbation of the onset of AD and related dementias, but not causally.	[231,232,233,234,235,236]
Diabetes, type 2	Very strong association of AF with diabetic complications, and of diabetes increasing the risk of AF.	[237,238,239,240,241,242,243,244,245]
Parkinson’s	Some evidence of an association of AF with early PD, much less so if PD is diagnosed later (i.e., evidence either way is relatively weak).	[246,247,248,249]
Rheumatoid arthritis	Small, significant association, but confounded with use of small molecule drugs. Also associated with greater risk of cardiovascular disease.	[250] [251] [252,253]

**Table 5:** Some markers that are raised in AF and that have been related to the genesis or presence of fibrinaloid microclots and coagulopathies in general.

Biochemical Marker	Comments	Selected References
Ferritin	Serum ferritin is a marker of cell death [188], whose accompanying release of free iron can cause microclots and may itself be induced by them or other traumas. It is significantly raised in AF.	[332,333]
Fibrinogen	Fibrinogen (including γ′ [334]) levels are commonly raised in inflammatory diseases [335]. Fibrinogen levels are higher in individuals with AF (and in those having a higher CHA_2_DS_2_-VASc score and likelihood of stroke), consistent with a role of microclots in the onset of AF.	[336,337,338,339,340,341,342,343,344,345]
	Fibrin clot properties also relate to stroke likelihood/severity in AF, though no amyloid measurements have yet been made.	[346,347]
Inflammation	Occurs (by definition) in all kinds of chronic, inflammatory disease [101], but is certainly associated with AF. An accompaniment to all syndromes involving microclots.	[343,348,349]
Plasminogen Activator Inhibitor-1 (PAI-1)	Significantly raised in AF, potentially reducing the rate at which fibrinaloid microclots might be removed.	[350,351,352]
Platelet Factor-4 and platelet activation	Platelet activation is another key feature of chronic, inflammatory diseases accompanied by microclots.	[353,354,355]
β-Thromboglobulin	Raised in AF.	[353,356,357]
Troponin (cardiac isoforms)	Probably more a metric of severity of cardiac events, but as a measure of cell death (like ferritin), it may have predictive value.	[358,359,360,361,362,363]
Von Willebrand Factor	It should be noted that it is unlikely to be a simple function, as too much or too little can be bad in terms of causing hyper- or hypo-coagulation, respectively [364].	[341,365,366]
